# Bilateral nervus intermedius neuralgia with neurovascular contact: a case report highlighting hypertension and eczema as potential precipitating factors

**DOI:** 10.3389/fpain.2026.1862625

**Published:** 2026-08-18

**Authors:** Shusheng Jiao, Yi Zhou, Yanxia Zhu, Zhekai Zhang, Haoyuan Ma

**Affiliations:** 1 Department of Neurology, Bethune International Peace Hospital, Shijiazhuang, Hebei, China; 2 Bethune International Peace Hospital, Shijiazhuang, Hebei, China

**Keywords:** bilateral, eczema, hypertension, nervus intermedius neuralgia (NIN), neurovascular contact, pregabalin

## Abstract

**Background:**

Nervus intermedius neuralgia (NIN) is a rare craniofacial pain syndrome. Bilateral involvement is extremely uncommon, and the pathophysiology and precipitating factors of NIN remain poorly understood.

**Case presentation:**

A 57-year-old female presented with a 3-month history of bilateral paroxysmal stabbing deep ear pain triggered by cold wind and auricular pressure, superimposed on a persistent dull ache. She had a 5-year history of hypertension, with poor blood pressure control (140–160/90–105 mmHg) for four months preceding symptom onset. Otoscopic examination revealed bilateral external auditory canal eczema. Dedicated cranial MRI demonstrated bilateral anterior inferior cerebellar artery (AICA) loops in contact with the facial-vestibulocochlear nerve complexes, with no vascular contact at the trigeminal or glossopharyngeal nerves. A clinical diagnosis of bilateral NIN was established according to ICHD-3 criteria. The patient responded favorably to pregabalin combined with strict blood pressure control. At three-month follow-up, her stabbing pain had essentially resolved (VAS: 0–1/10).

**Conclusion:**

This first case of bilateral NIN with radiologically confirmed bilateral neurovascular contact supports neurovascular conflict as a pathophysiological mechanism even in bilateral presentations. Uncontrolled hypertension and local inflammation may serve as potential precipitating factors. A conservative approach combining pharmacotherapy with cardiovascular risk factor management may achieve favorable outcomes, potentially avoiding surgery in selected patients.

## Introduction

Nervus intermedius neuralgia (NIN), also referred to as geniculate neuralgia, is an exceptionally rare craniofacial pain syndrome attributed to dysfunction of the nervus intermedius—a small sensory-motor branch of the facial nerve (cranial nerve VII) that courses between the facial and vestibulocochlear nerves before merging with the facial nerve within the internal auditory canal (1). Clinically, NIN is characterized by paroxysmal, sharp, or lancinating pain deep within the ear, which may radiate to the auditory canal, auricle, mastoid process, soft palate, or jaw angle, and is often triggered by tactile stimuli or mechanical manipulation of the ear region (2). Despite being defined by the International Classification of Headache Disorders, 3rd edition (ICHD-3), NIN remains a diagnostic challenge due to overlapping sensory innervation of the ear with the trigeminal (cranial nerve V) and glossopharyngeal (cranial nerve IX) nerves, leading to frequent misdiagnosis as trigeminal neuralgia or glossopharyngeal neuralgia (3). Moreover, its clinical presentation frequently deviates from ICHD-3 criteria, with reports of constant dull pain, absent trigger zones, and variable pain phenotypes, further complicating recognition (2).

The pathogenesis of NIN is not fully understood; however, neurovascular conflict is widely regarded as the primary etiological mechanism. This typically involves neurovascular contact involving the nervus intermedius or the facial- vestibulocochlear nerve complex (VII/VIII complex) by a vascular loop, most commonly the anterior inferior cerebellar artery (AICA) (4). The clinical effectiveness of current surgical interventions—namely microvascular decompression (MVD) and nervus intermedius sectioning—provides strong, practical validation for the NVC hypothesis (5, 6). MVD, which directly addresses the compressive pathology, achieves immediate pain relief in over 90% of patients with confirmed intraoperative NVC (7). Similarly, nervus intermedius sectioning, whether performed alone or in conjunction with MVD for concomitant cranial neuralgias, results in favorable short-term pain relief in approximately 80% of cases (5).

Despite some advances in surgical intervention, NIN remains poorly characterized due to its extreme rarity. The existing literature has predominantly focused on describing clinical manifestations and evaluating surgical outcomes (2, 3, 8). However, the specific precipitating or exacerbating factors for pain paroxysms—particularly in patients with established neurovascular contact—are not well delineated. Furthermore, bilateral NIN is exceptionally rare, with only two documented cases in the literature to date (9, 10), leaving the pathophysiological basis for bilateral disease incompletely understood.

We herein report a distinctive case of bilateral NIN in a 57-year-old female, which may provide several novel insights. First, dedicated cranial MRI confirmed bilateral NVC at the VII/VIII nerve complex, thereby substantiating neurovascular conflict as a plausible pathophysiological mechanism even in bilateral presentations. More notably, this case is distinguished by two clinically observable potential precipitating factors: (1) poorly controlled hypertension immediately preceding symptom onset, a systemic condition previously suggested to modulate neurovascular conflict in related conditions like trigeminal neuralgia (11); and (2) concurrent bilateral external auditory canal eczema, suggesting a possible role of localized inflammation in triggering or sensitizing nerve irritation. This combination of findings may offer a more integrated perspective on the multifactorial etiology of NIN. This case report was prepared in accordance with the CARE (CAse REport) guidelines.

## Case presentations

A 57-year-old female presented to the Otolaryngology clinic with a 3-month history of bilateral ear pain. The pain was characterized by a persistent, dull ache in both external auditory canals [visual analog scale (VAS) score 3–4/10], superimposed with recurrent, paroxysmal, sharp, and severe stabbing pain deep within the ears. The paroxysms were triggered by cold wind exposure to the ears and occasionally by pressure on the auricle. Notably, chewing, talking, swallowing, or face washing did not trigger the pain. Each stabbing pain episode had a VAS score of 8–9/10, lasted 3–10 s, and subsided, leaving behind a residual mild, dull ache in the external auditory canals. The patient reported no taste disturbances, obvious hearing loss, lacrimation, rhinorrhea, or salivary secretion abnormalities. Her past medical history was notable for a 5-year history of hypertension, which had been well-controlled on daily levamlodipine besylate 2.5 mg, with self-measured blood pressure (BP) consistently maintained at 125–135/70–85 mmHg. However, over the prior 4 months, she reported poor BP control (self-measured BP: 140–160/90–105 mmHg) attributed to work-related stress. She denied a history of type 2 diabetes, hyperlipidemia, coronary artery disease, chronic kidney disease, herpes zoster, trauma, or recent swimming.

**Figure 1:**
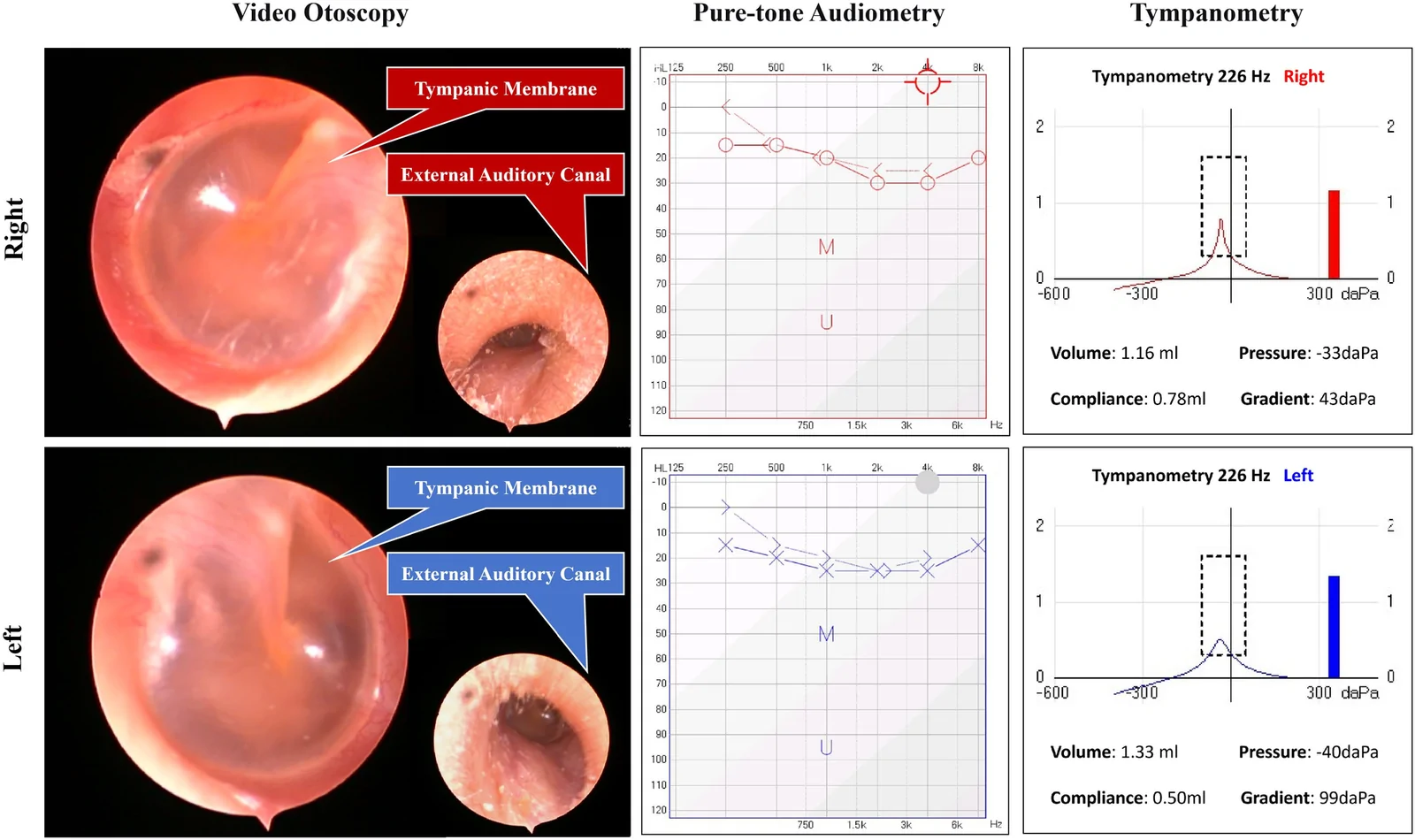
Findings of otolaryngological examinations. Otoscopic examination (right panel) demonstrated eczematous changes at the openings of both external auditory canals in the absence of any signs of herpes zoster. The ear canals were clear, and the tympanic membranes were intact with well-defined landmarks. Pure-tone audiometry (middle panel) showed bilateral mild high-frequency sensorineural hearing loss (right ear: 10 dB decrease at 2–4 kHz; left ear: 5 dB decrease at 1–4 kHz). Tympanometry (left panel) demonstrated bilateral type A tympanograms with normal compliance. dB, decibels; kHz, kilohertz.

In Otolaryngology clinic, initial physical and otolaryngology examinations were performed immediately. Vital signs showed a BP of 155/95 mmHg. General physical and systemic examinations were unremarkable. Neurological examination revealed an alert and cooperative condition with normal cranial nerve function, motor strength, sensation, reflexes, coordination, and no meningeal signs. Subsequent otoscopic examination showed eczematous changes at the openings of both external auditory canals, with no evidence of herpes zoster. The ear canals were patent, and the tympanic membranes appeared intact and well-demarcated (Figure 1, right panel). Pure-tone audiometry showed bilateral mild high-frequency sensorineural hearing loss (right ear: 10 dB decrease at 2–4 kHz; left ear: 5 dB decrease at 1–4 kHz) (Figure 1, middle panel). Tympanometry revealed type A curves bilaterally with normal compliance (right 0.78 mL, left 0.50 mL) (Figure 1, left panel). A non-contrast temporal bone MRI also showed no abnormalities. Laboratory tests including complete blood count, liver and renal function tests, blood glucose, and lipid profiles were all within normal limits. The otolaryngology team treated the eczema with topical ointments and referred the patient to Neurology department for further evaluation of the neuropathic pain.

**Figure 2:**
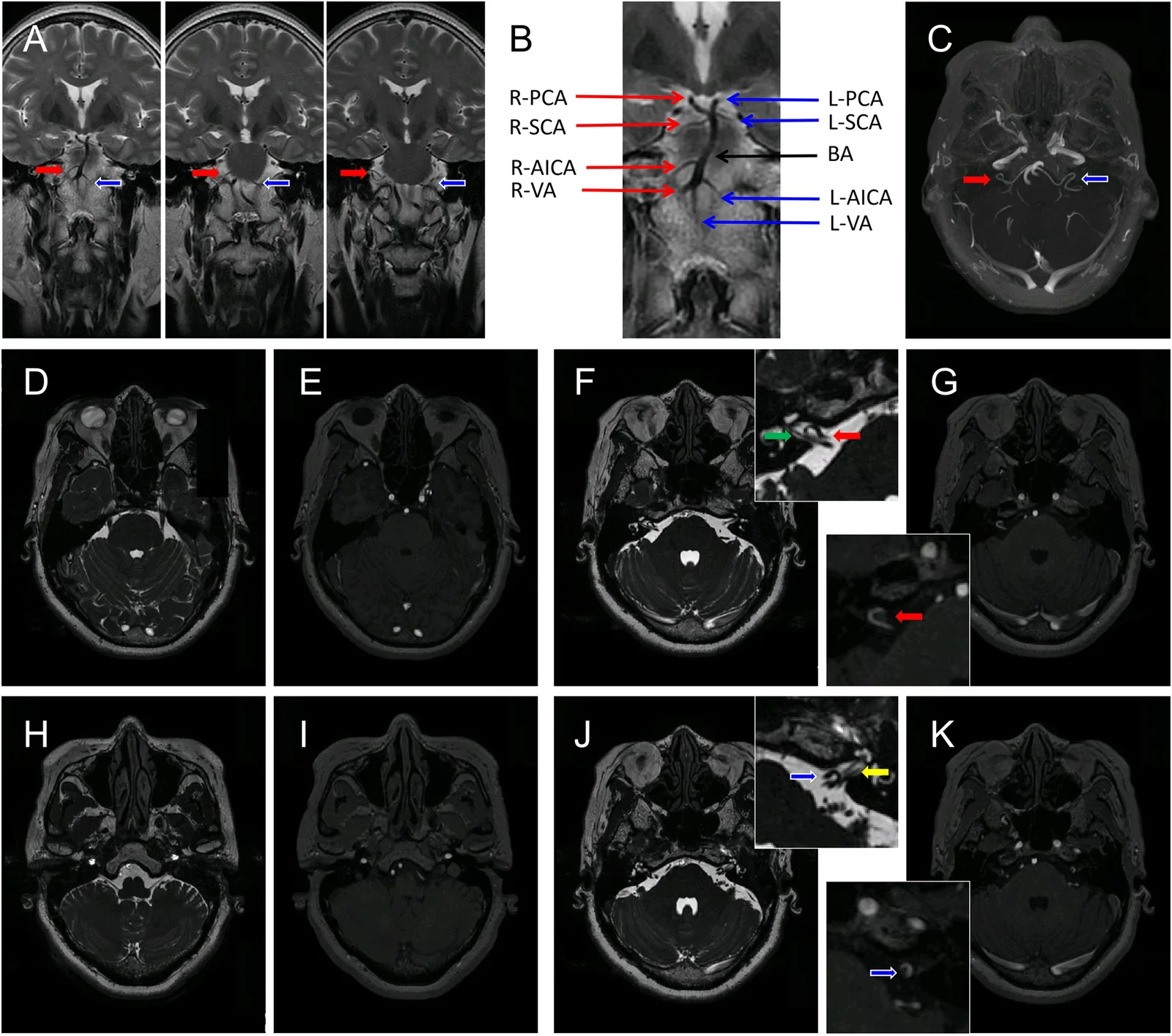
Findings of high-resolution cranial nerve MRI with ultra-thin slices. **(A)** Consecutive coronal thin-slice scans identified vascular loops at the cerebellopontine angle region arising from the bilateral anterior inferior cerebellar arteries (AICA). Red arrows indicate the course of the right AICA; blue arrows indicate the course of the left AICA. **(B)** Clear depiction of the major vascular distribution of the vertebrobasilar system at the brainstem. R-PCA, right posterior cerebral artery; L-PCA, left posterior cerebral artery; R-SCA, right superior cerebellar artery; L-SCA, left superior cerebellar artery; R-AICA, right anterior inferior cerebellar artery; L-AICA, left anterior inferior cerebellar artery; R-VA, right vertebral artery; L-VA, left vertebral artery. **(C)** 3D-TOF images demonstrate well-defined vascular loops formed by the bilateral AICA. **(D,F)** No definite vascular accompaniment or coiling is observed at the root entry zones of the trigeminal nerve. **(F,G)** The right AICA forms an accompanying vascular loop at the root entry zones of the right facial-vestibulocochlear nerve complex. Magnified images are placed in the upper inset [**(F)** 3D FIESTA-C sequence] and lower inset adjacent to the original image [**(G)** 3D-TOF sequence]. Red arrow indicates the right accompanying vascular loop; green arrow indicates the right facial-vestibulocochlear nerve complex. **(H,I)** No definite vascular accompaniment or coiling is observed at the root entry zones of the glossopharyngeal nerve. **(J,K)** The left AICA forms an accompanying vascular loop at the root entry zones of the left facial-vestibulocochlear nerve complex. Magnified images are placed in the upper inset [**(J)** 3D FIESTA-C sequence] and lower inset adjacent to the original image [**(K)** 3D-TOF sequence]. Blue arrow indicates the left accompanying vascular loop; yellow arrow indicates the left facial-vestibulocochlear nerve complex. FIESTA-C, fast imaging employing steady-state acquisition with cycled phases; 3D TOF, three-dimensional time-of-flight.

Upon referral to the Neurology department one week later, the patient's eczema had improved with topical treatment, and the persistent dull ache in both external auditory canals had partially resolved (VAS: 1–2/10). However, the characteristic paroxysmal, sharp, stabbing pain continued unabated, triggered identically by cold wind and auricular pressure. A dedicated cranial MRI with focused visualization of the facial nerve course was subsequently obtained. Serial section observation revealed vascular loops arising from the bilateral anterior inferior cerebellar arteries (AICA) accompanying and encircling the root entry zones of the bilateral facial and vestibulocochlear nerve complexes (Figures 2A–C–J,K). In contrast, no vascular accompaniment or coiling was observed at the root entry zones of the trigeminal (Figures 2D,E) or glossopharyngeal nerves (Figures 2H,I). Based on the classic clinical presentation—brief, severe, triggered, deep otalgia—and the correlative MRI evidence suggesting neurovascular contact, a clinical diagnosis of bilateral NIN was established in accordance with the ICHD-3 criteria. A comprehensive treatment plan was formulated, emphasizing strict blood pressure control and initiation of pharmacotherapy for neuropathic pain. Microvascular decompression surgery was reserved as a potential future intervention should medical management prove ineffective.

**Figure 3:**
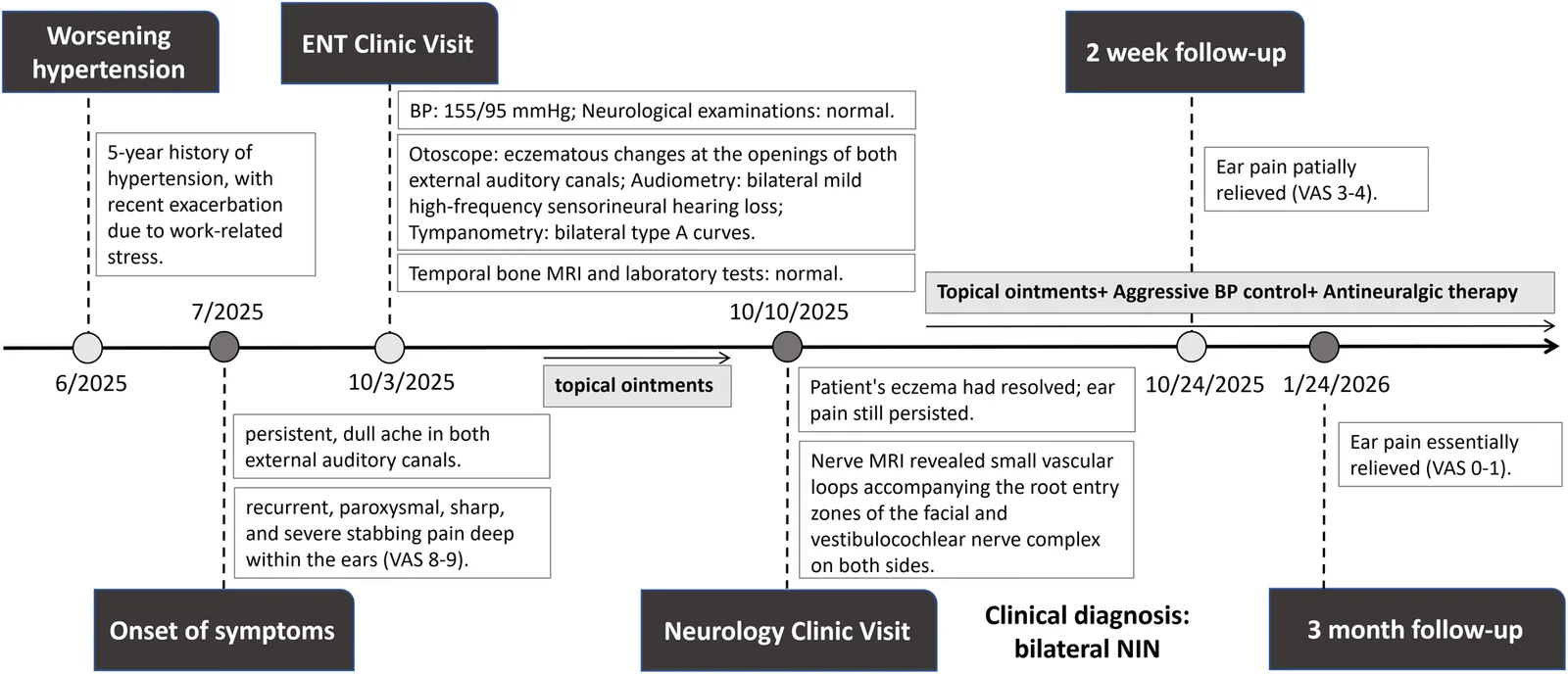
Timeline of the patient with clinical symptoms, diagnosis, and data before and after interventions. ENT clinic, ear, nose, and throat clinic; BP, blood pressure; NIN, nervus intermedius neuralgia; VAS, visual analog scale.

The patient declined first-line medications like carbamazepine and oxcarbazepine. Treatment was therefore initiated with the calcium channel modulator pregabalin at 75 mg twice daily, which was increased to 75 mg three times daily after three days. This was coupled with intensified management of her hypertension. At the 2-week follow-up, her blood pressure was well-controlled at 130/75 mmHg, and she reported significant clinical improvement: the dull background ache had completely resolved (VAS: 0/10), and the frequency and severity of the stabbing pain paroxysms were markedly reduced (VAS: 3–4/10). The pregabalin dosage was consolidated to 150 mg twice daily. After three month of sustained blood pressure control (125–135/70–85 mmHg) and continued medication, her stabbing deep-ear pain was essentially resolved (VAS: 0–1/10). The patient remains under ongoing follow-up. During this period, the patient has experienced occasional recurrence of deep bilateral ear pain associated with cold weather, but the pain intensity has remained mild (VAS: 3–4/10). The patient was satisfied with the improvement achieved with medical therapy and has consistently declined surgical options during follow-up. Figure 3 illustrates the patient's timeline, depicting the progression of clinical symptoms, diagnosis, and data collected before and after interventions.

## Discussion

This report describes a rare case of bilateral NIN in a 57-year-old female, characterized by radiologically confirmed bilateral neurovascular contact of the facial-vestibulocochlear (VII/VIII) nerve complexes, two potential precipitating factors (poorly controlled hypertension and bilateral external auditory canal eczema), and favorable response to conservative management with pregabalin and optimized blood pressure control. This case enriches the limited literature on bilateral NIN and may provide novel insights into its pathophysiology, triggers, and clinical management.

### Bilateral NIN: rarity and pathophysiological implications

NIN is an inherently rare condition, and bilateral involvement is exceptionally uncommon, with only two prior cases documented in the literature (9, 10). One case was managed with sequential bilateral nervus intermedius sectioning; notably, no neurovascular conflict was identified (9), leaving the pathogenesis of bilateral disease unresolved. In another reported case, surgical intervention involved sectioning of the nervus intermedius combined with MVD of cranial nerves VIII, IX, and X (10), raising questions about the definitive diagnosis of NIN vs. possible glossopharyngeal neuralgia. In contrast, our patient demonstrated clear bilateral neurovascular contact at the VII/VIII nerve complexes on dedicated cranial MRI, offering radiological support consistent with the neurovascular conflict hypothesis as a plausible pathophysiological mechanism for bilateral presentations. This finding aligns with the prevailing understanding that neurovascular contact—most commonly by AICA—is considered a primary etiology in unilateral NIN and suggests a possible extension of this established mechanism to its bilateral form. Nevertheless, it is important to interpret these imaging findings with appropriate caution, as neurovascular contact is not infrequently observed in asymptomatic individuals and does not, by itself, prove pathologic compression; in this case, the diagnosis was therefore established through integration of the MRI findings with the characteristic clinical phenotype and fulfillment of ICHD-3 criteria, rather than on imaging alone.

The presence of bilateral neurovascular contact in our patient points to a systemic or symmetric component in the underlying pathology. While the exact cause of bilateral vascular loop compression remains unclear, the broad reported age of onset for NIN—spanning from childhood (10, 12, 13) to a median of 45.5 years (5)—suggests that it may be related to congenital vascular anatomical variations or age-related vascular changes.

### Potential precipitating factors: hypertension and eczema

Beyond corroborating the structural basis of neurovascular contact, this case uniquely draws attention to two potential dynamic or peripheral modulating factors—poorly controlled systemic hypertension and bilateral external auditory canal eczema—which we propose as speculative precipitating contributors rather than established causes. Hypertension has been identified as a potential risk factor for other neurovascular compression syndromes (14) such as trigeminal neuralgia (11) and hemifacial spasm (15), presumably because elevated blood pressure increases vascular pulsatility and amplifies compressive forces on adjacent cranial nerves. However, its specific role in NIN onset has not been previously reported. In our patient, the temporal sequence is noteworthy: symptom onset followed a four-month period of uncontrolled hypertension (BP: 140–160/90–105 mmHg), and the resolution of lancinating pain paralleled effective blood pressure control (BP: 125–135/70–85 mmHg). While this chronological association is intriguing, it remains observational and does not prove causation, particularly given the concurrent initiation of pregabalin. A plausible—though speculative—pathophysiological link could involve increased vascular wall tension and pulsatility amplifying mechanical irritation of the nervus intermedius in a patient with pre-existing vascular contact, thereby potentially lowering the symptom threshold. In this hypothetical framework, systemic factors like hypertension might act as a “second hit” that facilitates symptomatic conversion in a predisposed anatomical configuration; however, this model requires validation through larger, controlled studies.

Similarly, to our knowledge, no published literature has directly linked external auditory canal eczema to NIN or other cranial neuralgias. Nonetheless, the bilateral nature and timing of the eczema in relation to the patient's otalgia raise a hypothesis-generating question about its potential contributory role. Anatomically, the nervus intermedius provides sensory innervation to the posteroinferior wall of the external auditory meatus and the inferior auricle. At the molecular level, in the setting of eczema, local inflammatory mediators—including cytokines, chemokines, and neurotrophic factors—released from activated keratinocytes and immune cells could theoretically act on peripheral nociceptive terminals (16), leading to reduced activation thresholds of transient receptor potential (TRP) channels, increased expression of voltage-gated sodium channels, and enhanced ectopic discharge from sensitized nerve endings (17). Clinically, the improvement in eczematous lesions with topical treatment was associated with partial resolution of the persistent dull ache in our patient. However, this temporal association is likewise inconclusive and may simply reflect concurrent natural history or placebo effects. Taken together, these observations generate the hypothesis that peripherally sensitized states, in a patient with pre-existing neurovascular contact, might lower the threshold for pain generation, converting a previously asymptomatic anatomical variant into a symptomatic one. We emphasize that this “dual-hit” model is purely speculative and serves primarily as a framework to guide future prospective investigations, rather than a definitive explanation of pathogenesis. Crucially, the absence of laboratory testing for varicella-zoster virus (VZV) antigens or antibodies in this case also precludes definitive exclusion of VZV-induced bilateral neuralgia, representing an alternative explanation that cannot be discounted.

### Conservative management: efficacy of pregabalin and blood pressure control

The favorable response to pharmacotherapy with pregabalin and rigorous blood pressure control in this case is also noteworthy. While MVD is often highlighted as the definitive treatment for NIN with neurovascular contact (7), our experience demonstrates that a tailored medical and systemic management strategy can yield excellent outcomes, potentially obviating the need for invasive surgery in some patients. Pregabalin, a calcium channel modulator, likely alleviated neuropathic pain by reducing neuronal hyperexcitability (18, 19). It must be acknowledged, however, that because pregabalin and blood pressure control were initiated concurrently, the observed clinical improvement cannot be definitively attributed to blood pressure normalization alone; pregabalin is the more established analgesic agent and likely contributed substantially to pain relief. Nonetheless, the significant symptom improvement temporally paralleling blood pressure normalization underscores the importance of evaluating and managing cardiovascular risk factors as an integral component of NIN treatment. This holistic approach—addressing both the neuropathic pain and its potential systemic amplifiers—may be key to effective long-term management.

### Differential diagnosis

The differential diagnosis for bilateral deep otalgia includes trigeminal neuralgia (V3 distribution), glossopharyngeal neuralgia, geniculate herpes zoster (Ramsay Hunt syndrome), temporomandibular joint dysfunction, and otologic conditions. In our patient, the absence of pain with chewing, talking, or swallowing made trigeminal (20) and glossopharyngeal neuralgias (21) less likely considerations. The absence of vesicular rash, facial palsy, along with normal otoscopy (apart from eczema), argued against Ramsay Hunt syndrome (22) and middle ear pathology (23). The normal temporal bone MRI and tympanometry tend to steer us away from structural otologic disease (24). The characteristic trigger points (cold wind, auricular pressure) and deep-ear localization, combined with dedicated cranial MRI demonstrating bilateral vascular contact at the VII/VIII complexes, supported the clinical diagnosis of NIN according to ICHD-3 criteria (25).

### Limitations

This case has several limitations. First, as a single case report, our findings cannot be generalized to all bilateral NIN patients. We cannot definitively establish causality between hypertension/eczema and NIN onset, only a temporal correlation. Second, MRI findings of vascular “contact” do not definitively prove “compression”, and the true anatomical existence of the nervus intermedius also remains to be established; both ultimately hinge on direct surgical observation. We acknowledge that the absence of clear radiological signs of nerve compression—such as displacement, flattening, or atrophy—limits the strength of the association between the observed vascular contact and the patient's symptoms. While the site-specific correlation with the VII/VIII complex and the fulfillment of ICHD-3 criteria support the diagnosis, the imaging findings alone cannot establish a definite causal relationship (26). Third, the concurrent initiation of pregabalin and blood pressure control confounds attribution of the clinical improvement to blood pressure normalization alone, as pregabalin is the more established analgesic agent. Fourth, long-term follow-up is needed to assess the durability of pain relief and the potential for recurrence. It is notable that eczematous changes were present in both external auditory canals prior to the onset of deep otalgia, which raised the possibility of herpetic neuralgia caused by varicella-zoster virus (VZV) reactivation. However, the absence of laboratory testing for VZV antigens or antibodies precludes definitive exclusion of a VZV-induced bilateral neuralgia in this case.

## Conclusion

This rare case of bilateral NIN with radiologically confirmed bilateral neurovascular contact expands our understanding of the pathophysiology and clinical spectrum of this enigmatic condition. It is compatible with neurovascular contact as a possible structural contributor in bilateral cases and proposes a dynamic, multi-hit pathophysiology potentially involving systemic hemodynamics and local inflammation. Nevertheless, the temporal association between blood pressure control and symptom improvement, while intriguing, should be interpreted with caution given the concurrent initiation of pregabalin. Notwithstanding this limitation, our findings emphasize that comprehensive management of NIN should extend beyond symptomatic pain relief to include the optimized management of modifiable risk factors such as hypertension. Clinicians should maintain a high index of suspicion for NIN in patients with bilateral deep otalgia, and utilize dedicated imaging and comprehensive evaluation of potential precipitating factors to guide individualized treatment. Most importantly, further studies—including larger case series and prospective cohorts—are needed to validate these findings, clarify the role of modifiable triggers, and refine treatment guidelines for NIN.

## Data Availability

The original contributions presented in the study are included in the article/Supplementary Material, further inquiries can be directed to the corresponding authors.
